# Hospitalization of Children Aged <5 Years Due to Influenza: Study Based on the National Hospitalization Registry

**DOI:** 10.3390/children9070930

**Published:** 2022-06-21

**Authors:** Krzysztof Kanecki, Katarzyna Lewtak, Paweł Goryński, Piotr Tyszko, Magdalena Bogdan, Michał Rząd, Aneta Nitsch-Osuch

**Affiliations:** 1 Department of Social Medicine and Public Health, Medical University of Warsaw, 3 Oczki Street, 02-007 Warsaw, Poland; kkanecki@wum.edu.pl (K.K.); ptyszko@wum.edu.pl (P.T.); mbogdan@wum.edu.pl (M.B.); michal.rzad@wum.edu.pl (M.R.); anitsch@wum.edu.pl (A.N.-O.); 2 National Institute of Public Health NIH—National Research Institute, 24 Chocimska Street, 00-791 Warsaw, Poland; pawel@pzh.gov.pl; 3 Institute of Rural Health in Lublin, 2 Jaczewskiego Street, 20-090 Lublin, Poland

**Keywords:** influenza, infectious disease, hospitalization, morbidity, nationwide registers, gender differences, region differences

## Abstract

Background: Influenza constitutes a significant health care burden that can be related to an increased morbidity and mortality. The aim of the study is to show up-to-date information on influenza hospitalization cases in Poland in children aged <5 years. Methods: The data used in the study were hospitalization records from 2015–2019, which had been gathered in a Nationwide General Hospital Morbidity Study and made available by the National Institute of Public Health. Results: 8565 records of patients with influenza were subject to analysis. The mean and median age of the patients were 2.2 and 2.1 years, respectively. Influenza hospitalization incidence was estimated at 90 per 100,000 person-years (PY) in children aged <5 years. The number of hospitalization cases was significantly higher in patients living in urban regions than in those living in rural regions (97 vs. 77 per 100,000 PY; *p* < 0.001). The age of patients living in urban regions was significantly lower than the age of patients living in rural regions (2.2 vs. 2.3 years; *p* < 0.001). A statistically significant increase in hospitalization was observed. Conclusions: The reported increasing trend in hospitalizations indicates the need to take focused measures. Presented data may be useful in comparative analyses in a European context.

## 1. Introduction

Annual hospitalizations in children aged under 5 have been estimated at 15 million worldwide [1]. According to the World Health Organization, the US Centers for Disease Control and Prevention, and other global health institutions, every year as many as 650,000 deaths can be associated with seasonal influenza [2].

Global annual respiratory deaths related to influenza corresponded to ~2% of all respiratory deaths recorded per year [3]. In 2018, the influenza virus globally accounted for 7% of acute lower respiratory infection (ALRI) cases, 5% of ALRI hospital admissions, and 4% of ALRI deaths in children under 5 years. Of these, about 23% of hospital admissions and 36% of in-hospital deaths were found in infants under 6 months. As many as 82% of in-hospital deaths were observed in low-income and lower-middle-income countries [4].

Another study showed that influenza can be related to a considerable burden of outpatient visits and hospitalizations in children <5 years of age [5]. With regard to the territory, the rate of influenza-related hospitalizations was three times lower in the industrialized countries than in the developing countries [6]. In a report from the Unites States, the cumulative hospitalization rate per 100,000 population was 46.9 for the 0–4 years age group for the period 1 October 2013–30 April 2014 [7]. In a study based on data from 16 countries, which represented 79% of the population of America, the average pooled rate of influenza-related respiratory hospitalizations in children under 5 years of age amounted to 90 cases per 100,000 population [8]. A study based on the Norwegian Patient Registry showed the highest hospitalization rates in children aged under 6 months and a drop in the number of hospitalization cases the older the children were [9]. A systematic review and meta-analysis made in China showed the highest rates of hospitalization related to influenza in the group of children at the age of <5 years, and thus this group was considered to significantly contribute to influenza-related morbidity and mortality burden [10]. It has been revealed in other studies that influenza infection is the cause of a considerable number of hospital admissions among young children [9,11,12,13,14,15].

According to the National Institute of Public Health NIH—National Research Institute in Poland, the incidence of influenza and influenza-like illness in Poland in 2015–2019 reached 10,000–14,000 cases per 100,000 population per year [16]. The epidemiological report from the National Institute of Public Health NIH—National Research Institute in Poland revealed influenza incidence of 57 per 100,000 children aged 0–4 years in December 2020, which affected this group the most [17]. Considering the serological specificity of influenza viruses, it seems justified to conduct a study on the frequency of influenza-related hospitalizations in children aged below 5 years.

Poland implemented the influenza surveillance system called Sentinel in 2004. The SENTINEL Influenza Surveillance System allows one to monitor the course and dynamics of epidemic seasons in this country. Virological data tallied by 16 Voivodship Sanitary and Epidemiological Stations allow one to identify the dominant virus strain and to provide information on the age groups [18]. Over the last four influenza epidemic seasons, an increase in the percentage of confirmed influenza infections reported in the SENTINEL Surveillance System has been observed [19]. Another study focused on the clinical samples of pediatric patients aged 0–14 years that were obtained in the season of 2014/2015 epidemics in Poland. Virological infection was confirmed for 50.9% of samples, 38.2% of which were samples of children at the age of 0–4 years.

In the study, influenza viruses accounted for 48.0% and other respiratory viruses for 52.0% of all positive samples [20]. Previous population studies in Poland indicate that children aged 0–4 years are most likely to be exposed to influenza [21].

Vaccination is a very important way to prevent influenza virus infection and hospitalizations derived from complications caused. It is recommended by the WHO to perform annual vaccinations in children aged 6 months–5 years [22]. However, many children are still not vaccinated despite numerous vaccination recommendations, and thus the influenza burden remains [23]. According to a report from the United States, many pregnant women reject vaccinations despite the protective nature of these for the mother and the infant [24]. Diagnostic tests for influenza are important as they allow one to confirm the presence of flu and help implement focused treatment. Over 10 years, a sharp rise from 3.2% to 42.4% was observed in Poland with regard to the relation between the influenza laboratory confirmations across seasons and the number of specimens tested [25].

The highest influenza burden can be observed in infants, which is also reflected in the economic burden [26].

Considering the importance of influenza burden, it is highly necessary to collect recent data on influenza hospitalizations in Poland among children aged <5 years based on data from national morbidity register. These data may be a basis for comparative analyses in a European context.

## 2. Materials and Methods

The study is a retrospective, population-based study carried out with the use of hospital discharge records of children aged below 5 years and diagnosed with influenza. The data used in the study were 8565 records of patients with influenza in the years 2015–2019 that had been gathered in a Nationwide General Hospital Morbidity Study and made available by the National Institute of Public Health NIH-National Research Institute in Poland. In the study, influenza-related hospitalization records, with both primary and secondary influenza diagnosis, were analyzed. All Polish hospitals, except for psychiatric facilities, are legally bound to provide hospital discharge data to the Institute. These are anonymous data on patient’s sex, date of birth, and place of residence; hospital admission and discharge data; and the code of the International Classification of Diseases (ICD-10) assigned during the diagnosis.

Comorbidities were divided into groups according to the ICD-10 classification. Although the data were anonymous, the available information was the basis for an approximate analysis of the frequency of hospitalization for individual cases. The study was reported to the local bioethics committee. It was assumed that influenza had been diagnosed in hospitals according to the most up-to-date and commonly used criteria. However, in our database no information was given directly on the laboratory confirmation of influenza. Patients were included in this study if hospitalized with an ICD-10 code of ‘J10—influenza due to identified influenza virus’ or ‘J11—influenza due to unidentified influenza virus’. Hospitalization cases with an ICD-10 code of J09 were not observed. A similar method of data collecting was used in Norway. The Norwegian Patient Registry (NPR) collects clinical data obtained from all Norwegian hospitals. It is obligatory for hospitals in that country to report ICD-10 codes of diagnoses to the NPR [27].

To perform the statistical analyses, Statistica (TIBCO Software Inc., Palo Alto, CA, USA) [28] and WINPEPI [29] were used. The following statistical measures were computed: means, medians, and ranges for continuous variables, and counts and percentages for categorical variables. For continuous variables with normal or non-normal distribution, respectively, means and 95% confidence intervals or medians were calculated. For nominal variables, counts and percentages were analyzed. Rates of hospitalizations related to influenza infection were calculated as the estimated number of unique patients per 100,000 person-years in children aged <5 years using data (national census) from the Central Statistical Office of Poland [30]. To assess trends, we used linear regression. Analyses were performed using the t-Student test with respect to an assumption of normal distribution in sufficiently large samples in public health research [31]. When normality assumptions were not met, non-parametric tests (Chi-square, U Mann–Whitney) were applied. A two-sided p-value less than 0.05 was considered to be statistically significant.

## 3. Results

**Figure 1:**
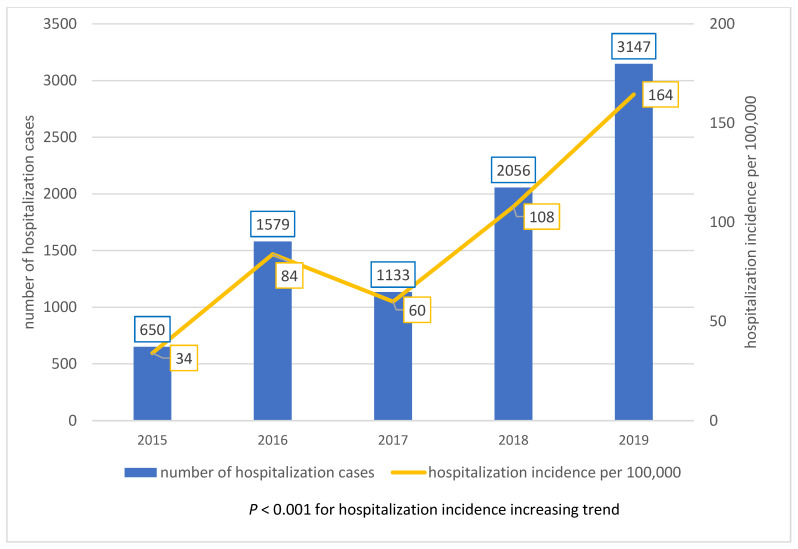
The number of influenza hospitalization cases, 2015–2019.

In the study group, we analyzed 8565 hospitalization records of influenza hospitalizations. ICD-10 code of ‘J10—Influenza due to other identified influenza virus’ was reported in 83% of cases in this study. On the basis of the available data, it was estimated that in 95% of cases, patients were hospitalized only once in the analyzed period of time. Patients from the study group were hospitalized in the pediatric ward (47%), hospital emergency ward (13%), children observation and infectious ward (10%), or neonatology ward (7%), and others separately (below 5%). The study group consisted of 4702 males (55% of all patients) and 3863 females (45% of all patients). A significant predominance of male patients was observed among hospitalized patients in relation to the analyzed population (55% vs. 51%; *p* < 0.001). In the study group, the mean and median age were 2.2 and 2.1 years. There were no significant differences in age between males and females. Presented in Figure 1, a statistically significant increase in hospitalization was observed in the study (*p* < 0.05, adjusted R2 squared = 0.76). In the study population, the number of hospitalizations for children under 1 year of age increased nominally, but its percentage decreased significantly (*p* < 0.005), from 283 cases in 2015 (44% of all hospitalizations) to 723 (23% of all hospitalization in 2019).

**Figure 2:**
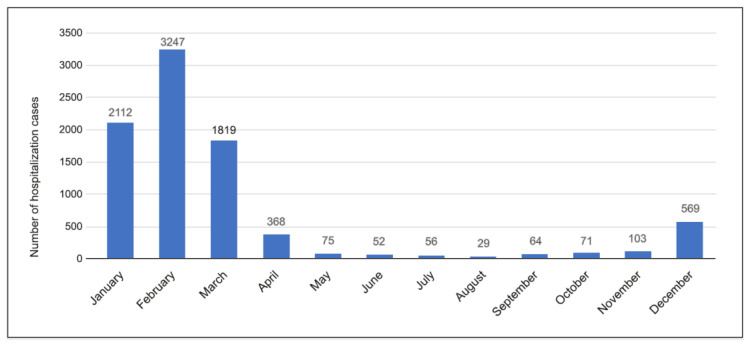
Number of influenza hospitalization cases per month.

In this study, a seasonal pattern was found over the observational period with the highest rates of hospitalizations in January–March period in all years, and the lowest in August, as presented in Figure 2.

**Figure 3:**
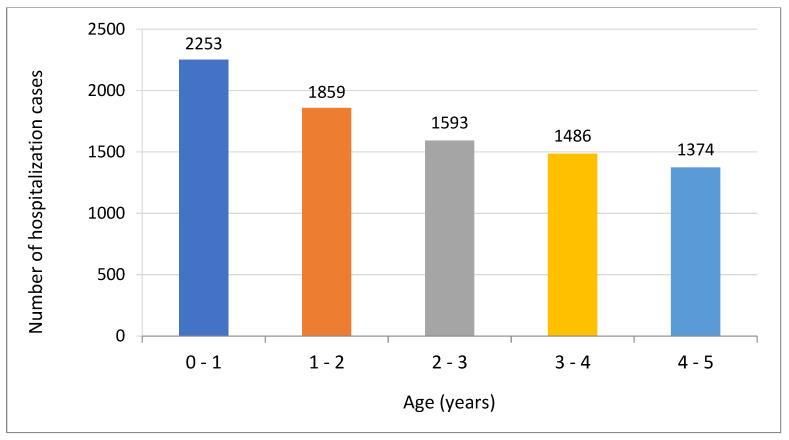
Number of influenza hospitalization cases per children age.

As reported in Figure 3, most of the cases were observed in children under 1 year of age. At this age, we observed 2253 cases (26% of all cases). Additionally, the hospitalization rate at the level of 16% of all hospitalizations occurred in the <6-month-age-group.

In our study, mean and median hospitalization rate for patients with influenza were estimated to be 90 per 100,000 person-years. The number of hospitalizations was significantly higher in patients living in urban regions than in patients living in rural regions (97 per 100,000 person-years vs. 77 per 100,000 person years; *p* < 0.001). There was no information on the place of residence in 137 cases (1.6% of all hospitalizations). The age of patients living in urban regions was significantly lower than the age of patients living in rural regions (2.2 vs. 2.3 years; *p* < 0.001). 

The following comorbidities were the most common in the study group: respiratory system-related diseases other than influenza (26.5% of patients), followed by certain infectious and parasitic diseases (13.4%), diseases of the eye and adnexa (9.3%), endocrine, nutritional and metabolic diseases (5% of patients), and other groups of diseases (below 5%). Among these groups of diseases, respiratory-system-related disease other than influenza was significantly more often observed in males than females (27.5% vs. 25.3%; *p* < 0.05).

During the observation period, 9 deaths were reported (0.1% of all patients). Among them 5 cases were males, 4 females. Mean and median age in this subgroup were 1.5 years (95% CI: 0.6–2.4) and 1.3 years (interquartile range: 0.67–1.76). In urban regions we observed 5 cases, in rural regions 4 cases.

## 4. Discussion

In a study analyzing hospitalizations in all age groups due to influenza-related infections of the lower respiratory tract, 123.8 cases per 100,000 population worldwide, 386.8 per 100,000 in Central Europe, and 55.7 per 100,000 in Poland were estimated [32]. Data from our study based on hospitalization registers reported the incidence of 90 per 100,000 person-years in children below 5 years of age. However, it should be mentioned that influenza hospitalizations only were taken into account in the estimation. The Centers for Disease Control and Prevention in the United States monitor hospitalizations related to laboratory-confirmed influenza infections using the Influenza Hospitalization Surveillance Network. One study from the US reported the cumulative hospitalization rate of 72.0 per 100,000 population of children at the age of 0–4 years [33]. In our study, the highest hospitalization rate occurred in the <1-year-age-group. In a study from Singapore, the highest influenza-related hospitalization rates were observed in the group of infants <6 months [15]. In another study from Norway, the highest hospitalization rates were reported in patients aged 80 years and more, and in children under 5 years of age [27]. A retrospective study based on the annually collected data on confirmed influenza cases in 1998–2014 in selected hospitalized children aged 0–17 years in Stockholm, Sweden showed the highest admission rate of 169 per 100,000 in the group of children <3 months of age [34].

A study based on The Norwegian Patient Registry revealed a decrease in the hospitalization rates among children with influenza the older the children were [9]. A similar trend was observed in our study. However, there is an increasing trend for the number of influenza-related hospitalizations in the study group, which is shown in Figure 1.

The increase in the frequency of hospitalizations in the studied group of patients, more pronounced in children above 1 year of age, observed in 2015–2019 may result from the generally growing trend of influenza and influenza-like illness in Poland in 2014–2018, according to data reported by the National Institute of Public Health NIH-National Research Institute in Poland [35].

The reason of the increasing trend observed in our study may be multifactorial and related to the efficiency of health care, changes in the virulence of the virus, the health policy of the state regarding compulsory vaccination, or other unknown factors. It is necessary to continue the research in this field in order to reveal the sources of this disturbing trend. Immunization as a way of limiting this trend should be given special attention.

In one study based on data from 2011 to 2016 obtained in Suzhou, China, high hospitalization rates of influenza-associated severe acute respiratory infections were observed in children under 5 years of age. The study showed that vaccinating children >6 months of age as well as mothers and caregivers of those <6 months could lead to the reduction of hospitalizations related to influenza in the group of young children in Suzhou [36]. This report from China is also important due to our observations presented in Figure 3. In our study, most hospitalization cases were observed in children under 1 year of age. Seasonality in hospitalizations, which took place mainly in the winter and spring months, as presented in Figure 2, is similar to the seasonality of influenza in the general population, with the highest incidence of influenza in the period January–March [35]. In comparison with the 2018/19 epidemic season in the United States, the activity of influenza-like illness started to increase in November, reached a peak in mid-February, and dropped to below baseline values in mid-April [33]. In this study, the significant predominance of male patients was observed. Such observations may suggest a more aggressive course of influenza in these patients, which requires hospitalization. In a systematic analysis based on 89 studies on severe acute infections of the lower respiratory system that resulted in hospital admissions of young children around the world, it was observed that the incidence was higher in the group of boys than in the group of girls, and the biggest sex disparity was shown in the studies from South Asia [1].

The male gender may also be a potential risk factor for influenza-related hospitalizations among patients under 5 years of age in Poland. In our study, other factors may also be related to hospitalization. The number of hospitalization cases was significantly higher in patients living in urban regions than in those living in rural regions, and the age of patients from the former group was significantly lower that the age of the latter group. Influenza, which is mainly transmitted by droplets, may spread more slowly in rural settings probably due to lower population density, possible greater social distancing, or less mobility of the population. In a study from China, significant differences were reported between influenza-like illness cases among children aged 0–4 years with regard to urban and rural areas of Shenyang in 2010–2018. In this age group, the highest incidence of influenza-like illness cases was observed (46.90% for urban and 49.43% for rural areas) [37]. The risk of hospitalization for influenza can be increased by the pre-existing risk conditions. However, no pre-existing risk conditions were observed in the majority of children (75%) who were admitted to hospital in Norway with regard to influenza in the years 2017–2019 [9].

In our study, diseases of the respiratory system other than influenza were the most common comorbidities in the hospitalized patients and were significantly more often observed in male than female patients. A large, population-based cohort study from Australia revealed a substantial healthcare burden related to influenza in children suffering from a range of chronic lung diseases [38]. In another study from Australia, a total of 1268 cases of children hospitalized with influenza were analyzed, and comorbidities predisposing to severe influenza were found in 45.1% of patients [39]. In our study group, there were 9 cases of death, which accounts for 0.5 per 100,000 hospitalizations. Another study showed the following ratios for deaths related to influenza and infections of the lower respiratory tract: 1.9 per 100,000 worldwide, 3.9 per 100,000 in Central Europe, and 1.4 per 100,000 in Poland [32]. The percentage of fatal hospitalizations in our study group seems to be low and may probably be related to the estimation based only on hospitalizations.

However, a systematic review and meta-analysis showed that as many as 28,000–111,500 children aged <5 years died in 2008 because of influenza-related acute infections of the lower respiratory system, and a vast majority of these deaths (99%) were observed in the developing countries [11].

It should be mentioned that the data from this study can be helpful in making national decisions to reduce risk of influenza complications. One of the most important ways to reduce the risk is to use preventive vaccinations, with special attention paid to pregnant women. The following risk reductions were observed in children born to women vaccinated against influenza during pregnancy: 64% for influenza-like illness, 70% for laboratory-confirmed influenza, and 81% for influenza hospitalizations in the first 6 months of life [40]. No link was found between vaccinating women against influenza during pregnancy and adverse birth outcomes [41]. As shown in another study, an important preventive measure is to perform influenza vaccinations among household contacts of infants aged under six months [42]. It was observed that performance of influenza vaccinations in mothers was related to the fall in the number of hospital admissions for acute respiratory diseases in infants aged under 6 months [43]. The result of our study suggested age, sex, comorbidities, place of living, and season as risk factors for influenza hospitalization in children under 5 years of age. Further focused research is necessary to carry out a better analysis of these factors.

According to the data of the National Institute of Public Health NIH–National Research Institute, in 2020 23,153 children aged 0–4 years were vaccinated, and the number of vaccinated people in the Polish population in all age groups was 1,046,633 [44]. In Poland, flu vaccinations for children are recommended, not obligatory, and paid for by the patient. The data presented in this study, and in particular the growing trend of hospitalization of children aged 0–4 years in recent years, suggests the need to take urgent measures regarding the use of preventive vaccinations against influenza in Poland. Our study has certain limitations. General information on hospitalizations was obtained from the database. However, this database did not allow for a broad examination of individual risk factors and other correlates in patients. What is more, there was no pre-assessment of the ICD-10 coding practices. According to the guidelines for the ICD-10 codes, the influenza virus requires identification. Influenza tests are easily accessible, and there are no limitations on their use. However, many patients are not tested or they are tested too late to confirm the diagnosis, and therefore they might be admitted to hospital with other diagnoses. For this reason, there is a possibility of influenza underestimation, which might lead to imprecise data on the number of incident cases. On the other hand, however, the considerable length of the observation period and the size of the database that was built on the National Register of Hospital Morbidity are of great benefit to this study.

## 5. Conclusions

Influenza hospitalization incidence among children aged <5 years in Poland can be related to age, sex, comorbidities, seasons, and territorial differences. The increasing trend in hospitalizations in recent years, which was observed in the study, indicates the urgent need to take focused measures. We suggest that interventions, such as vaccination, should be combined with mask wearing, social distance, hand hygiene, and focused changes in organization of health care. These actions may reduce the overall burden of influenza and outcomes among children in Poland. This work covers the period before the COVID-19 pandemic and therefore could be the basis for further comparative analyses in the future with regard to the influenza data in the pandemic period. Presented data may also be useful in comparative analyses in a European context.

## Data Availability

Nationwide General Hospital Morbidity Study, National Institute of Public Health NIH—National Research Institute, Warsaw, Poland.
